# Relationship between Symptoms and Gene Expression Induced by the Infection of Three Strains of *Rice dwarf virus*

**DOI:** 10.1371/journal.pone.0018094

**Published:** 2011-03-22

**Authors:** Kouji Satoh, Takumi Shimizu, Hiroaki Kondoh, Akihiro Hiraguri, Takahide Sasaya, Il-Ryong Choi, Toshihiro Omura, Shoshi Kikuchi

**Affiliations:** 1 Research Team for Vector-borne Plant Pathogens, National Agricultural Research Center, Tsukuba, Ibaraki, Japan; 2 Division of Genome and Biodiversity Research, National Institute of Agrobiological Sciences, Tsukuba, Ibaraki, Japan; 3 Plant Breeding, Genetics, and Biotechnology Division, International Rice Research Institute, Metro Manila, Philippines; Institut Pasteur, France

## Abstract

**Background:**

*Rice dwarf virus* (RDV) is the causal agent of rice dwarf disease, which often results in severe yield losses of rice in East Asian countries. The disease symptoms are stunted growth, chlorotic specks on leaves, and delayed and incomplete panicle exsertion. Three RDV strains, O, D84, and S, were reported. RDV-S causes the most severe symptoms, whereas RDV-O causes the mildest. Twenty amino acid substitutions were found in 10 of 12 virus proteins among three RDV strains.

**Methodology/Principal Findings:**

We analyzed the gene expression of rice in response to infection with the three RDV strains using a 60-mer oligonucleotide microarray to examine the relationship between symptom severity and gene responses. The number of differentially expressed genes (DEGs) upon the infection of RDV-O, -D84, and -S was 1985, 3782, and 6726, respectively, showing a correlation between the number of DEGs and symptom severity. Many DEGs were related to defense, stress response, and development and morphogenesis processes. For defense and stress response processes, gene silencing-related genes were activated by RDV infection and the degree of activation was similar among plants infected with the three RDV strains. Genes for hormone-regulated defense systems were also activated by RDV infection, and the degree of activation seemed to be correlated with the concentration of RDV in plants. Some development and morphogenesis processes were suppressed by RDV infection, but the degree of suppression was not correlated well with the RDV concentration.

**Conclusions/Significance:**

Gene responses to RDV infection were regulated differently depending on the gene groups regulated and the strains infecting. It seems that symptom severity is associated with the degree of gene response in defense-related and development- and morphogenesis-related processes. The titer levels of RDV in plants and the amino acid substitutions in RDV proteins could be involved in regulating such gene responses.

## Introduction

Virus interacts with host proteins, and disturbs the gene expression of host cell. Responses of plants at the gene expression level to various viruses were examined using microarrays to explore the molecular basis of symptom development and defense systems [1]–[8]. Comparison of results from previous studies indicated that, although many genes in various plant species respond specifically to different viruses, there is commonality in responses among different plant-virus interactions [1], [2]. Virus infection often results in the suppression of genes related to development and morphogenesis processes, and the suppression of such genes appears to cause disease symptoms, although, the individual genes within a group suppressed by virus infection vary depending on plant species and tissues, and virus species [2]–[6]. Virus infection also activates genes related to stress- and pathogenesis-related (PR) responses [1]–[8]. The induction of genes for these processes is related not only to defense against viruses, but also to abnormal plant development. The gene-silencing process is one of the major virus defense systems [9]–[12]. Suppression of genes involved in the gene-silencing process may cause abnormal plant development [10], [12]. Activation of PR genes also causes abnormal plant growth [13]–[15].

Rice dwarf disease limits rice production in East Asian countries. Rice plants affected by the disease show symptoms such as stunted growth, chlorotic specks on leaves, and delayed and incomplete panicle exsertion [16]. Rice dwarf disease is caused by *Rice dwarf virus* (RDV). RDV is transmitted to rice plants by insects, in particular leafhoppers (*Nephotettix* spp.), after multiplication of the virus in the insect. Many cell wall- and chloroplast- related genes were suppressed, whereas various defense-related genes were activated in rice plants infected with RDV [7].

Three strains of RDV differentiated by the severity of symptoms they cause were reported [17]. Rice plants infected with the severe strain of RDV (RDV-S) were significantly more stunted than those with the ordinary strain of RDV (RDV-D84). Another strain, RDV-O, originating from RDV-D84, causes weaker symptoms than those caused by RDV-D84. To reveal specific gene responses associated with the difference in symptom severity caused by different RDV strains, we compared the gene responses in rice individually infected with RDV-S, -D84, and -O using a 60-mer oligonucleotide microarray. The result indicated that the gene responses to RDV infection were regulated differently depending on the gene group and RDV strains, and that symptom severity is associated with the degree of gene response in defense-related and development- and morphogenesis-related processes.

## Results

### 1. Characterization of three RDV strains

**Figure 1:**
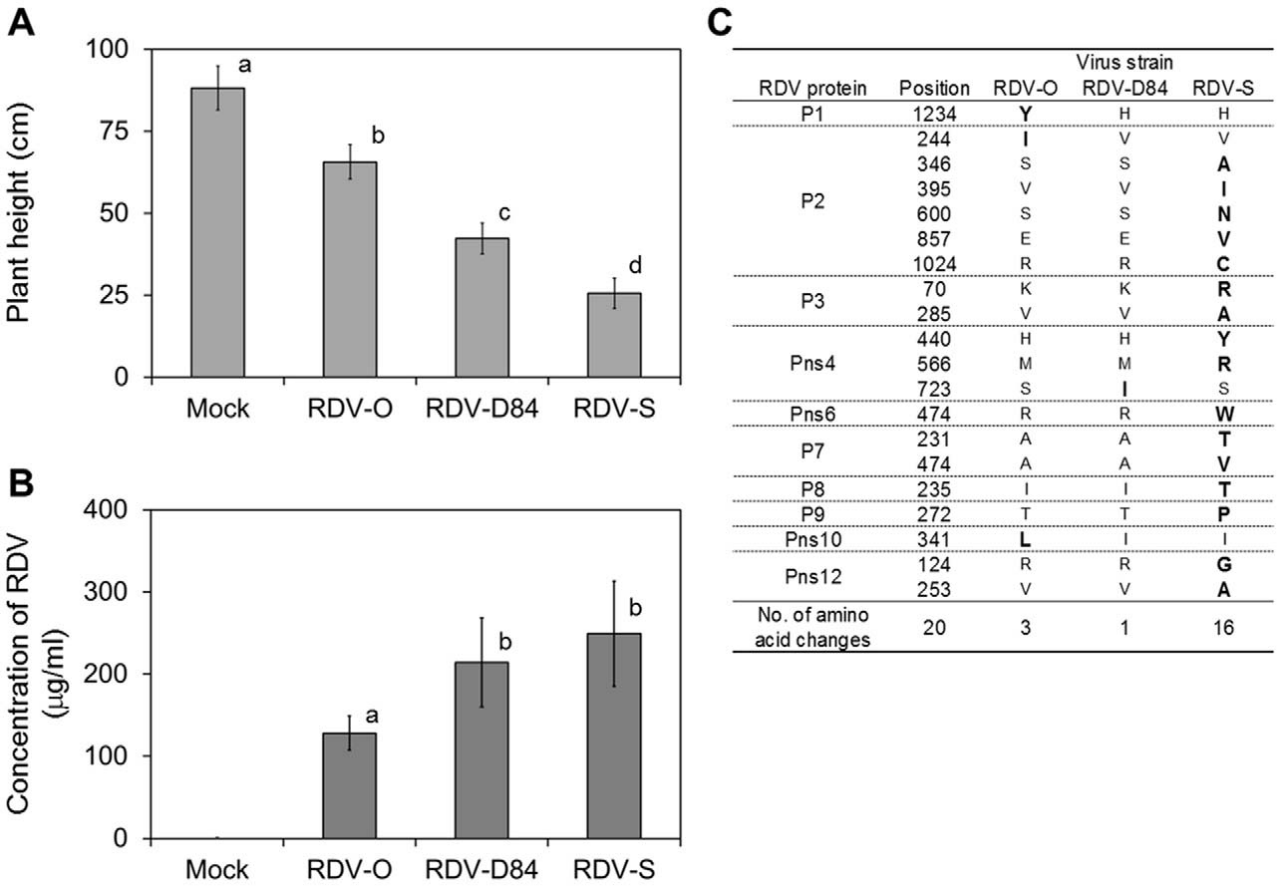
Characterization of three RDV strains. A): Heights of plants infected with three RDV strains at 40 dpi. Common letters are not signficantly different at the 1% level by least significant difference test. Vertical lines indicate standard deviation. B): Concentrations of RDV strains at 30 dpi estimated by enzyme-linked immunosorbent assay. Vertical lines indicate standard deviation. Common letters are not significantly different at the 5% level by least significant difference test. Vertical lines indicate standard deviation. C): Amino acid substitutions of three RDV strains.

RDV strains RDV-O, -D84 and -S were independently inoculated into 11-day-old rice seedlings by viruliferous green leafhopper (GLH: *Nephotettix cincticeps*). At 8 days post inoculation (dpi), disease symptoms such as stunting and leaf stripes were observed and the differences in symptoms caused by the respective RDV strains became distinct after 18 dpi. The plants inoculated with virus-free GLH (mock-inoculated plants) did not show any symptoms (Figure 1A, and Supplementary Figure S1). The height of plants infected with RDV was significantly shorter than that of mock-inoculated plant. The height of the plants infected with RDV-S was lowest, and that of plants infected with RDV-O was highest (Figure 1A, Supplementary Table S1A).The titer of RDV in the infected plants was also different among plants infected with different RDV strains (Figure 1B). The concentrations of RDV-D84 and -S were significantly higher than that of RDV-O, but the concentrations of RDV-D84 and -S were not significantly different (Figure 1B).

The entire genome sequences of the three RDV strains were determined (Supplementary Table S1B). Twenty amino acid substitutions were found among the proteins encoded in the genomes of the three RDV strains (Figure 1C, Supplementary Table S1C). Sixteen amino acid substitutions were specific to the proteins encoded in the genomes of RDV-S. RDV-S-specific amino acid substitutions were found in eight proteins (P2, P3, Pns4, Pns6, P7, P8, P9, and Pns12, Figure 1C). Three amino acid substitutions were specific to RDV-O (P1, P2, and Pns10, Figure 1C).

### 2. Transcriptome analysis

**Figure 2:**
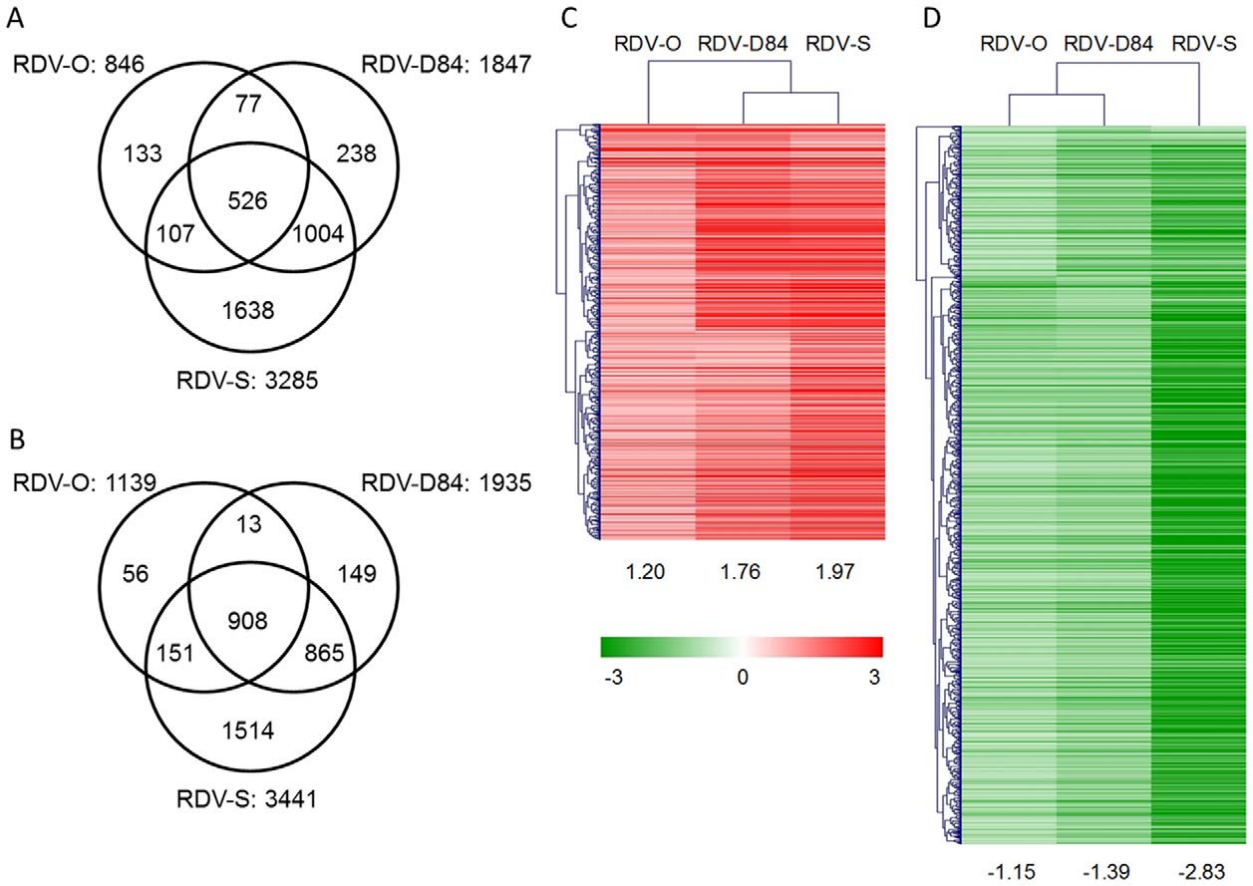
Numbers of specific and common differentially expressed genes (DEGs), and hierarchical clustering of common DEGs among plants infected with three RDV strains. A): Activated DEGs. B): Suppressed DEGs. The number of common activated and suppressed DEGs is 526 and 908. Hierarchical clustering of common activated (C) and suppressed DEGs (D) by Pearson correlation was performed by Mev ver. 4.4 [69]. The numbers under the heatmaps are the average log_2_ ratios of common DEGs in plants infected with the respective RDV strains.

To elucidate the basis of differences in symptom severity caused by three RDV strains at the gene expression level, we compared gene expression profiles among plants infected with the respective RDV strains using a 60-mer oligonucleotide microarray. Gene expression changes in response to RDV infection were detected by direct comparison between mock- and RDV-inoculated plants. The number of differentially expressed genes (DEGs) was different among plants infected with three RDV strains. The number of DEGs in plants infected with RDV-O, -D84, and -S was 1985, 3782, and 6726, respectively (Figure 2A and B, Supplementary Table S2). To assess the accuracy of microarray data, we selected 17 DEGs and two non-DEGs and examined the similarity between gene responses observed by microarray and those by RT-PCR. Most cases of activation or suppression of gene expression detected by microarray were also observed by RT-PCR, although the degree of the response was different for some genes (Supplementary Figure S2).

The individual DEGs induced by three RDV strains were similar (Figure 2A and B). About 90% of the DEGs by RDV-O infection also showed a response in rice plants infected with RDV-D84 and/or -S, and more than 90% of the DEGs by RDV-D84 infection also showed a response in plants infected with RDV-O and/or -S (Figure 2A and B). The numbers of commonly activated and suppressed DEGs among plants infected with the three RDV strains were 526 and 908, respectively (Figure 2A and B). A hierarchical clustering analysis of the common DEGs indicated that the degree of their responses varied among plants infected with the three RDV strains (Figure 2C and D). Generally, the degree of gene response to RDV-O infection was lowest and the response to RDV-S infection was highest. The degree of gene activation by RDV-D84 infection was closer to that by RDV-S infection than to that by RDV-O infection, whereas the degree of gene suppression by RDV-D84 was closer to that by RDV-O infection.

### Defense- and stress-related genes

**Figure 3:**
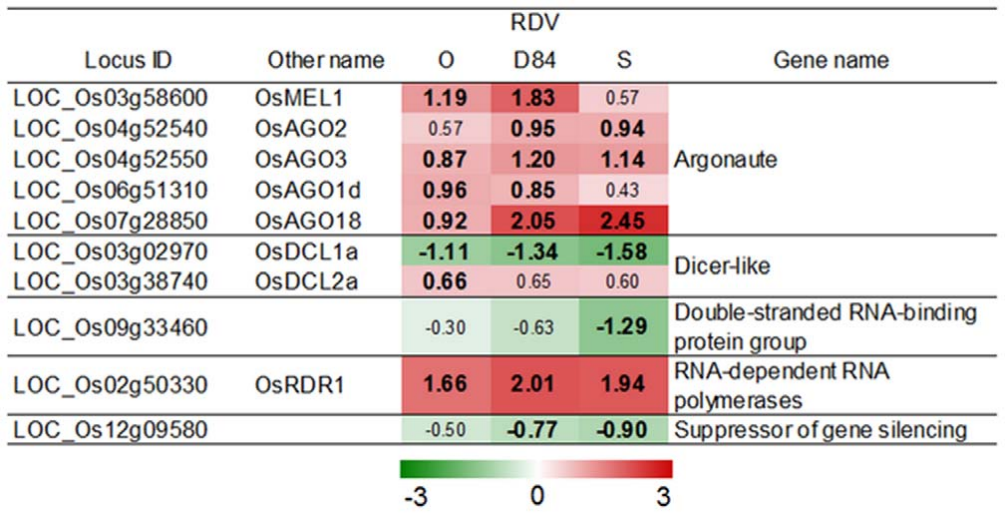
Responses of genes related to gene-silencing systems by RDV infection. The log_2_-based differential expression ratios (signal intensity in RDV-infected plant/signal intensity in mock-inoculated plant) of genes after infection with RDV strains are indicated by green (suppressed) or red (activated) colors of various intensities. Only the ratios of genes that were declared as a DEG in at least in one plant by an RDV strain are shown. Numbers in bold are the differential expression ratios of genes declared as a DEG (see Materials and Methods).

One of the host defense systems against virus infection is the gene-silencing system. The expression of the genes involved in the gene silencing system is often activated by virus infection [9]. Several genes for argonaute protein and RNA-dependent RNA polymerase, which are involved in the production of small interfering RNA [9], [10], [18], were also activated by RDV infection (Figure 3). The degree of activation of genes for the gene-silencing system by RDV infection was similar among plants infected with the three RDV strains (Figure 3).

**Figure 4 pone-0018094-g004:**
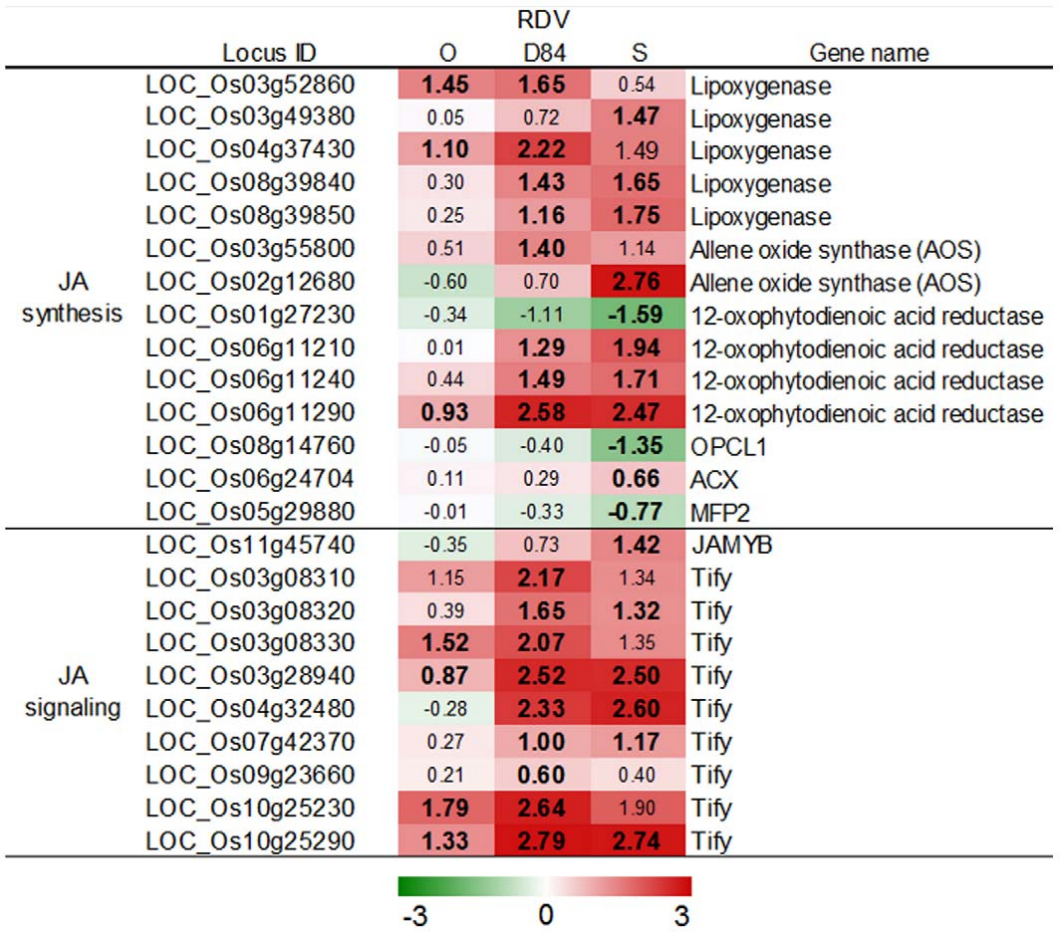
Response of genes related to JA synthesis and signaling processes to RDV infection. See Figure 3 for details.

**Figure 5 pone-0018094-g005:**
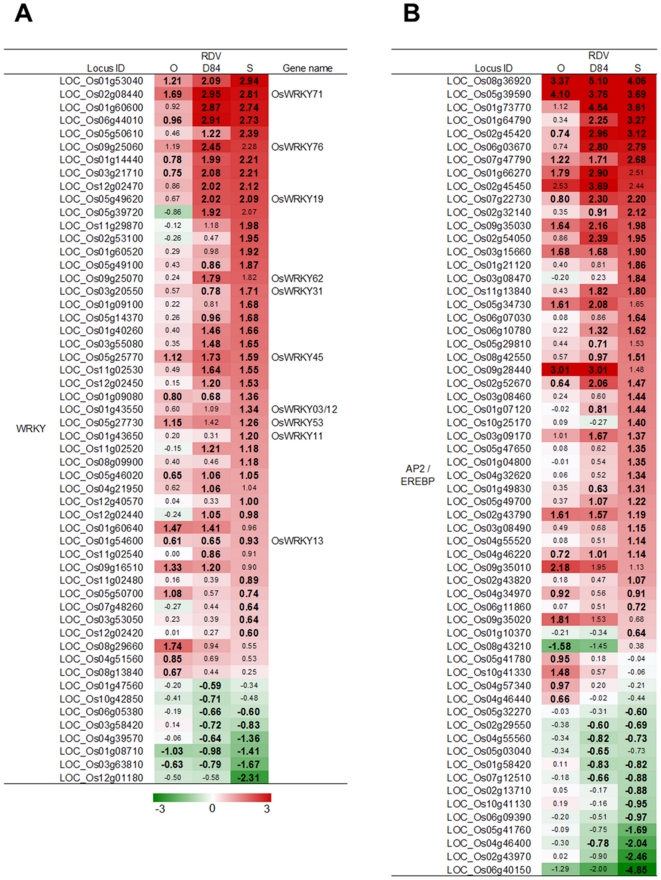
Response of genes belonging to WRKY and AP2/EREBP families to RDV infection. A): WRKY family. B): AP2/EREBP family. See Figure 3 for details.

Plant hormone-regulated systems are also involved in defense against virus infection [19], [20]. The genes for jasmonic acid (JA) synthesis were induced by RDV infection. Especially, genes for enzymes involved in the early steps in JA synthesis such as lipoxygenase and allene oxide synthase were highly activated (Figure 4). Tify family and *JAMyb* genes encode JA-responsive transcription factors [21], [22]. The expression of genes for Tify family and *JAMyb* was also activated by RDV infection (Figure 4). The number and degree of activation for DEGs in plants infected with RDV-O were less than in plants infected with RDV-D84 and -S. However, the number and degree in plants infected with RDV-D84 were similar to those in plants infected with RDV-S (Figure 5). Ethylene (ET) and salicylic acid (SA) are also involved in hormonal defense systems [19], [20], [23]. However, the genes for ET and SA synthesis were not strongly activated by RDV infection (Supplementary Table S2).

**Figure 6:**
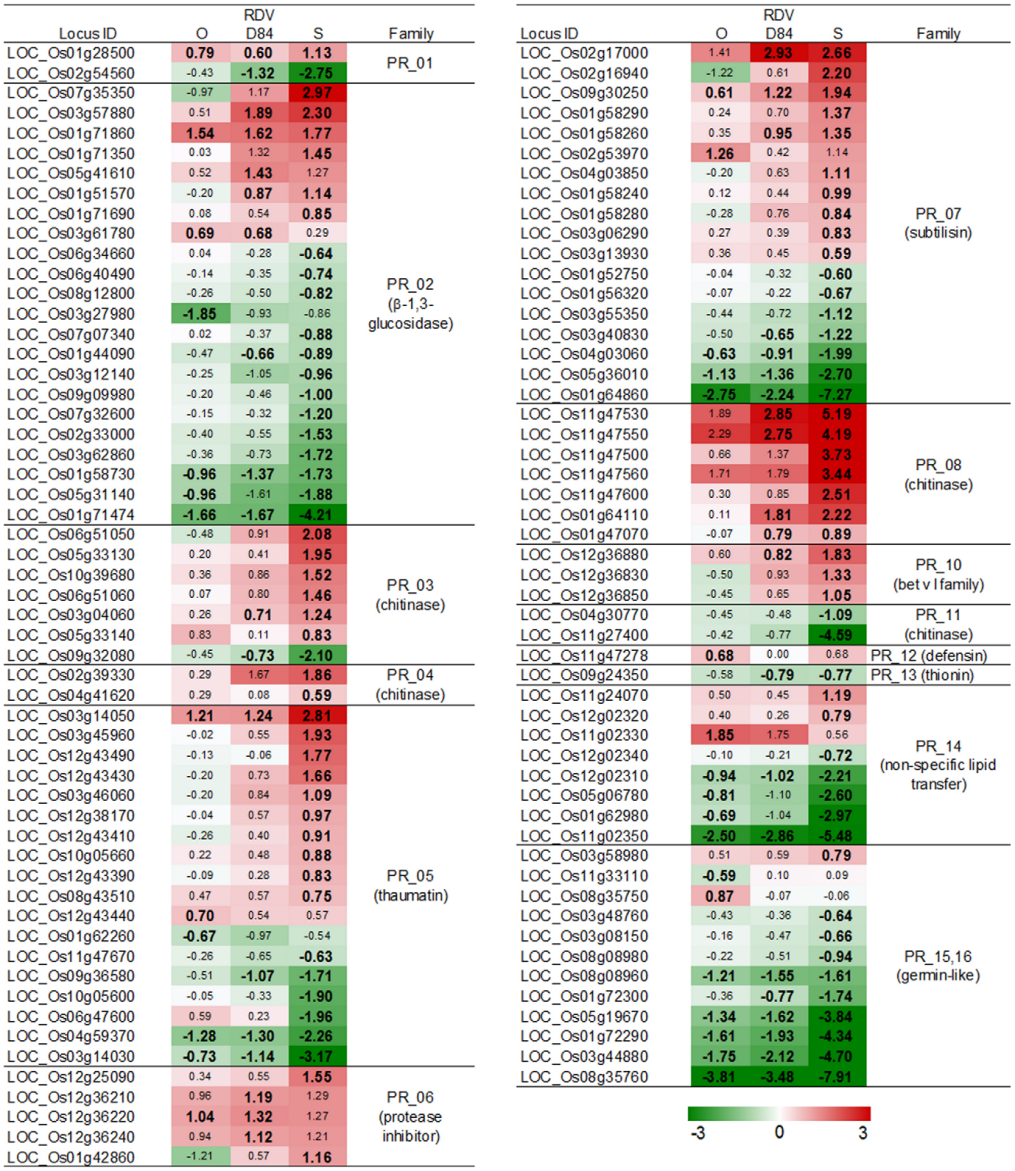
Response of pathogenesis related gene families to RDV infection. See Figure 3 for details.

Hormone-regulated defense systems are controlled by transcription factors such as WRKY and AP2/EREBP (named from APETALA 2/ETHYLENE RESPONSIVE ELEMENT BINDING PROTEIN) families [24]–[26]. Many WRKY and AP2/EREBP genes were activated by RDV infection (Figure 5A and B). The genes regulated by WRKY and AP2/EREBP include PR protein genes [24], [25]. PR proteins are classified into several types according to their biochemical functions [27]. The expression of PR protein genes was changed by RDV infection. The direction of the gene response was different from the type of PR protein (Figure 6). Many genes for PR1 (SCP-like extracellular domain-containing proteins), chitinases (PR3, 4, and 8), PR5 (thaumatin-like proteins), PR6 (protease inhibitors), and PR10 (pathogenesis-related Bet v I family proteins) were activated, whereas the genes for PR2 (β-1,3-glucosidases), PR14 (non-specific lipid transfer proteins), and PR15 and 16 (germin-like proteins) were predominantly suppressed by RDV infection (Figure 6). The expression of other defense- and stress-related genes such as those for glutathione S-transferases (GST) and heat shock factors was also activated by RDV infection (Supplementary Figure S3). The number and degree of response for DEGs associated with defense and stress response processes by RDV infection were different among plants infected with the three RDV strains. For activated DEGs, the degree of response by RDV-D84 infection was generally higher than that by RDV-O infection, but was similar to that by RDV-S infection (Figure 5 and 6). For a majority of suppressed DEGs, the degree of response by RDV-D84 infection was similar to that by RDV-O infection, but was lower than that by RDV-S infection (Figure 6).

### Development- and morphogenesis-related genes

**Figure 7:**
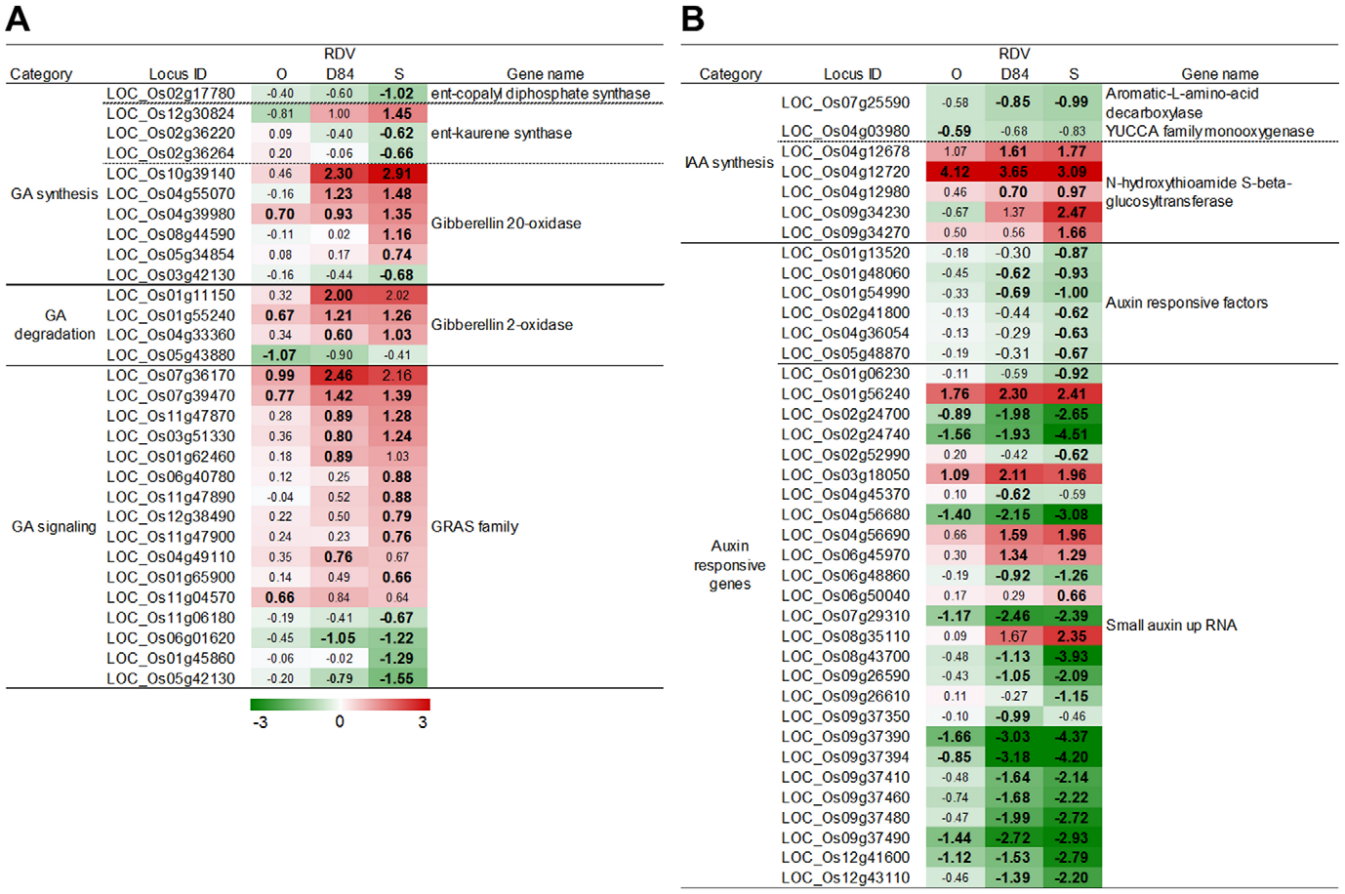
Response of genes related to GA and IAA synthesis and signaling processes to RDV infection. A): GA synthesis and signaling. B): IAA synthesis and signaling. See Figure 3 for details.

Development and morphogenesis processes are often controlled by plant hormones. Gibberellic acid (GA) is a plant hormone that promotes shoot elongation. Genes involved in early reaction of GA synthesis such as those for *ent*-kaurene synthase [28] were suppressed by RDV infection, whereas genes involved in GA inactivation processes such as those for gibberellin-2-oxidase [28] were activated (Figure 7A). Genes belonging to the GRAS (named from “GIBBERELLIC ACID-INSENSITIVE,” “REPRESSOR of GAI,” and “SCARECROW”) family encode negative regulators of GA signaling [29]. RDV infection activated expression of the GRAS gene family (Figure 7A). The responses of genes related to GA synthesis and signaling were similar between the plants infected with RDV-D84 and RDV-S, whereas the genes encoding *ent*-kaurene synthase were suppressed only in plants infected with RDV-S.

Indole acetic acid (IAA) is a plant hormone involved in development processes such as shoot elongation. Genes for aromatic-L-amino-acid decarboxylase and YUCCA family monooxygenase, which are involved in the early steps of IAA synthesis [30], were suppressed by RDV infection (Figure 7B). Six genes for auxin response factor (ARF), which is a positive regulator of auxin signaling [31], were suppressed (Figure 7B). Many auxin-responding SAUR (SMALL AUXIN UP RNA) [32] genes were also suppressed by RDV infection (Figure 7B). The degree of suppression for genes related to IAA synthesis and signaling was highest in plants infected with RDV-S and lowest in plants infected with RDV-O (Figure 7B).

**Figure 8:**
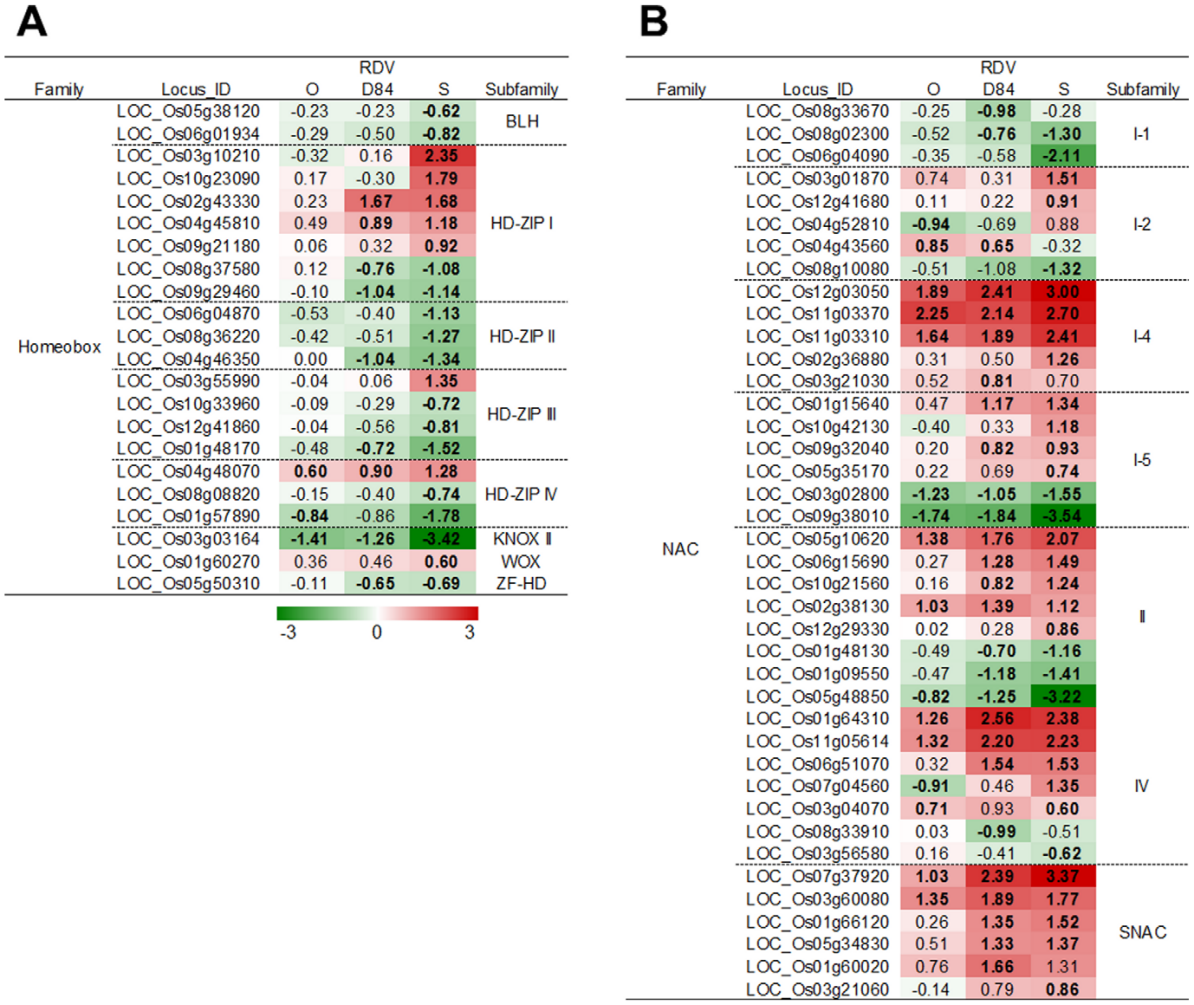
Response of genes related to auxin synthesis and signaling processes to RDV infection. A): Homeobox family. B): NAC family. See Figure 3 for details.

Various transcription factors are closely regulated during development and morphogenesis processes [33]–[41]. The homeobox gene family is associated with the development and morphogenesis of plants [33], [34]. The expression of many HD-zip-type homeobox genes was suppressed by RDV infection, except for the genes classified in HD-zip I, which were predominantly activated by RDV infection (Figure 8A). Genes for other transcription factors involved in development processes were also suppressed by RDV infection (Supplementary Figure S4). In constract, the genes for many transcription factors categorized into NAC (named from “NAM,” “ATAF1,” and “CUC2”) and DOF (DNA-BINDING WITH ONE FINGER) were activated by RDV infection (Figure 8B, Supplementary Figure S4) [41]. The degree of response for genes for these transcription factors was dependent on the RDV strains infecting and the direction of the gene response. The degree of suppression by RDV-D84 infection was similar to that by RDV-O infection, and was less than that by RDV-S infection, whereas the degree of activation by RDV-D84 was higher than that by RDV-O infection and was similar to that by RDV-S infection.

Our previous study showed that RDV infection suppresses the expression of genes related to cell wall and chloroplast formation [2]. The current study also showed the suppression of genes related to cell wall formation such as those for cellulose synthases and arabinogalactan proteins (Supplementary Figure S5). The degree of suppression of cell wall-related genes in plants infected with RDV-O was similar to that in plants infected with RDV-D84, but it was lower than in plants infected with RDV-S. In contrast, many genes for wall-associated kinases, which bind to pectin [42], were activated by RDV infection (Supplementary Figure S5). The degree of activation for wall-associated kinase genes in plants infected with RDV-D84 was similar to that in plants infected with RDV-S, but it was higher than in plants infected with RDV-O (Supplementary Figure S5).

Many genes associated with photosynthesis, carbon fixation processes, and chlorophyll synthesis were suppressed by RDV infection in this study (Supplementary Figure S6). Genes associated with chlorophyll degradation were not activated by RDV infection. The gene response was also different among plants infected with the three RDV strains. The genes involved in photosynthesis pathway were usually suppressed mostly only in plants infected with RDV-S. In carbon fixation and chlorophyll metabolism, many genes were suppressed in plants infected with RDV-D84 and RDV-S. Only a few genes such as those for ribulose-bisphosphate carboxylase and cytochrome c6 were also suppressed in plants infected with RDV-O (Supplementary Figure S6).

## Discussion

Three RDV strains caused disease symptoms such as stunting and chlorotic specks, but the severity of symptoms, especially stunting, varied among plants infected with the three strains (Figure 1A, and Supplementary Figure S1). The plants infected with RDV-S were most stunted and those infected with RDV-O were least stunted (Figure 1A). The RDV titer levels were also dependent on the RDV strains. The level of RDV-O was lowest, but the titer level of RDV-S was not significantly different from that of RDV-D84 (Figure 1B). This result implies that the severity of disease symptoms is not simply related to the level of RDV titer in infected plants, and that other factors may be involved in symptom development.

### 1. Defense- and stress response-related genes regulated by RDV infection

RDV infection activated the expression of many groups of genes associated with defense and stress response processes, although some genes were suppressed (Figures 3–6). The gene-silencing system is one of the important systems of defense against virus infection [9]. RDV infection activated many genes likely related to the RNAi process. *SHOOTLESS4* (*SHL4*) in rice is the gene encoding a component of the *trans*-acting siRNA process for endogenous genes, which is one of the post-transcriptional gene-silencing (PTGS) processes [10]. *Dicer-like 2* (*DCL2*) is involved in the PTGS process in *Arabidopsis* [18]. RNA-dependent RNA polymerase 2 (RDR2) works with DCL3 to form chromatin-associated siRNAs in *Arabidopsis* [18]. *RDR1* in *Arabidopsis* produces viral secondary siRNAs following viral RNA replication-triggered biogenesis of primary siRNAs [43]. In plants infected with RDV, rice genes that are likely orthologous to genes for *RDR1* and *DCL2* (*OsRDR1*: LOC_Os02g50330, *OsDCL2a*: LOC_Os03g38740, [44]) were activated by RDV infection. In addition, *OsAGO2* (LOC_Os04g52540) and *OsAGO3* (LOC_Os04g52550), which are paralogous genes of *SHL4* [44], were also activated by RDV infection. The expression of genes related to the gene-silencing process did not vary significantly among plants infected with the different RDV strains (Figure 3). These observations suggest that the difference in titer level among RDV strains is not associated with the expression of the genes for the gene-silencing process.

JA is a signal molecule for the regulation of a defense system against biotic stresses. The genes for JA synthesis and signaling were induced by RDV infection (Figure 4). *RIM1* (LOC_Os03g02800) is a NAC family gene, and a negative regulator of JA signaling [45]. RDV propagation was suppressed in a *rim1* mutant [46], whereas genes for JA synthesis and JA-mediated signaling were quickly and highly induced in the *rim1* mutant by wounding [45]. These observations suggest that JA-mediated defense systems in rice plants are involved in the suppression of RDV propagation. In this study, the *RIM1* gene was suppressed in the respective plants infected with RDV strains (Figure 8). The genes for JA synthesis and JA-mediated defense systems were highly induced in plants infected with RDV-S (Figure 4). This result suggests that the activation of defense systems controlled by JA after RDV infection may not be enough to inhibit propagation of RDV in plants expressing functional *RIM1*. The inconsistency between the result with the *rim1* mutant and this study indicates that quick induction of JA-mediated defense systems may be important for suppressing RDV propagation.

RDV infection also induced many types of genes related to biotic stress responses such as those encoding AP2-EREBP, WRKY, PR protein families, and wall-associated kinase (Figures 5 and 6, Supplementary Figure S5) [24]–[27], [42]. Expression of *WRKY45* gene (LOC_Os05g25770), which is reported to be induced by SA and not by JA [26], was increased by RDV infection, although the genes for SA synthesis were not induced by RDV infection (Supplementary Table S2). Thus, the defense systems regulated by *WRKY45* and SA signaling could also be induced by RDV infection.

### 2. About development and morphogenesis processes

Virus infection affects plant growth and development processes, and the disturbance of gene expression by virus infection may lead to the development of disease symptoms such as dwarfism and mosaic on leaves [1]–[8]. Genes related to cell wall and chloroplast functions were suppressed by RDV infection [7]. In this study, the suppression of these genes was observed in plants infected with three RDV strains (Supplementary Figures S5 and S6). The suppression of these genes was also observed in plants infected with other viruses [1], [3]–[6], [8]. Plants infected with *Plum pox virus*, *Tomato spotted wilt virus* and *Rice stripe virus* (RSV) showed symptoms such as dwarfism and chlorosis. Genes for cell wall and chloroplast functions were also suppressed in plants infected with these viruses [4], [6], [8]. Therefore, the suppression of these genes may be related to symptom development.

The suppression of GA and IAA synthesis and signaling processes was observed in plants infected with RDV (Figure 7). The suppression of GA related genes was also observed in stunted plants infected with RSV and *Soybean mosaic virus* [8], [47]. The loss of function in GA synthesis and signaling resulted in dwarfism in rice and *Arabidopsis* plants [48]–[51], and transgenic plant expressing genes for GA degradation showed the dwarfism [52]. ARF genes affect development in *Arabidopsis* and rice [53], [54]. A transgenic rice plant in which expression of the ARF1 gene was repressed exhibited development abnormalities such as stunted growth, short leaves, and delayed flowering [53]. These observations suggest that the suppression of GA and IAA synthesis and signaling is also associated with dwarfism caused by RDV infection.

Suppression of transcription factor genes such as those encoding homeobox, TCP, and SBP families resulted in abnormal development and growth [33]–[41]. In this study, HD-zip family genes responded to RDV infection. The many genes of HD-zip II, III and IV families were suppressed by RDV infection, whereas those of the HD-zip I family were induced. In *Arabidopsis*, the functions of HD-zip genes are dependent on the types of domain encoded in the genes [55]. The genes of HD-zip I are involved in stress responses and development, while HD-zip II genes are involved in auxin signaling and development. HD-zip III and IV function in development processes [55]. Therefore, the difference in responses among HD-zip gene families in plants infected with RDV may be associated with the gene functions dependent on domain types. NAC family genes are involved in the regulation of plant development and stress responses [41], [56]. The expression of many NAC genes was changed by RDV infection. Especially, some genes in SNAC (stress-responsive NAC, [56]) family were induced by RDV infection. Thus, like HD-zip genes, the responses of NAC genes seem to be dependent on the encoded domain types, which may be related to distinctive gene functions.

The activation of genes for defense processes affects plant development. The *rim1* mutant showed stunted shoot growth [46]. A high concentration of endogenous JA inhibited shoot growth [57]. Some genes for defense systems such as those for PR proteins are also associated with plant development and morphogenesis processes [14], [58]. Therefore, the activation of genes for defense processes may be related to symptom development.

### 3. The difference in gene responses by three RDV strains

Gene responses to RDV infection can be largely categorized into three types: 1) responses that are similar among all infected plants, independent of the RDV strain; 2) responses that are similar in plants infected with RDV-D84 and RDV-S; and 3) responses that are similar in plants infected with RDV-O and RDV-D84.

A Type 1 response is found in the genes for gene silencing. Virus genomes often encode a protein to inhibit the gene-silencing process in host cells (silencing suppressor) in order for viruses to propagate in host cells [9]. Pns10 in RDV functions as a suppressor of gene silencing processes in host cells [59]. One amino acid substitution was found in Pns10 of RDV-O (Figure 1C). A mutant of *Cucumber mosaic virus*, which does not express the silencing suppressor protein, accumulated at a low level in *Arabidopsis*, indicating that the mutation of the silencing suppressor affected virus propagation in plants [43]. These observations suggest that RDV titer levels may be related to the possible difference in protein structure of Pns10 among different RDV strains.

A Type 2 response is mainly found in genes activated by RDV infection (Figure 2C), such as genes involved in stress response and defense processes. It seems that the degree of response of genes in this category is correlated with RDV titer levels.

A Type 3 response is found in the expression patterns of development- and morphogenesis-related genes. It seems that a Type3 response may not be associated with RDV titer levels, since the degree of suppression in plants infected with RDV-D84 is lower than that with RDV-S, although the titer level in RDV-D84-infected plants was similar to that in RDV-S-infected plants. The suppression of host gene expression compared among *Nicotiana* plants infected with some RNA viruses such as *Cymbidium ringspot virus, Turnip crinkle virus, Ribgrass mosaic virus, and Cucumber mosaic virus* (CMV) showed that the severe suppression of host genes was associated with the development of severe symptoms [3]. The amino acid changes in virus proteins are also associated with the disease symptoms. Some virus proteins of *Tomato leaf curl virus* (TLCV) are associated with disease symptoms. Transgenic plants expressing mutated TLCV genes encoding C2, C3, C4, and V1 showed significantly milder symptoms than those expressing the wild type TLCV genes [60]. The symptom severity on *Nicotiana* plants infected with CMV was associated with the protein sequence of coat protein and not the level of the titer or gene product [61]. Therefore, the lack of association between RDV titer levels and Type 3 gene response may be due to the difference in amino acid sequences among different RDV strains. In RDV, seven structural (P1, P2, P3, P5, P7, P8, and P9) and five non-structural proteins (Pns4, Pns6, Pns10, Pns11 and Pns12) are encoded in the 12 genome segments of double stranded RNA [62]. Pns6 is localized to plasmodesmata and identified as necessary for cell-to-cell movement of RDV [63]. Pns10 functions as a suppressor of gene-silencing processes in host cells [59]. Sixteen amino acid substitutions in eight virus proteins were specific to RDV-S (Figure 1C), Five of 16 amino acid substitutions in RDV-S were found in P2 protein. P2 interacts with *ent*-kaurene oxidase and inhibits GA synthesis [64]. The response of genes involved in GA synthesis and the signaling process by RDV infection indicated that endogenous GA content may decrease in infected plants, and that the decrease may be more drastic in plants infected with RDV-S. Suppression of genes for GA synthesis and signaling could be associated with the difference in P2 protein sequences among RDV strains. In this study, we suggest that disease severity by RDV strains is dependent on the difference in expression of various genes, which is in turn associated with RDV titer level and the variations in virus proteins among RDV strains. In a further study, we would like to investigate the interaction between host and virus proteins to determine the mechanisms of symptom development by RDV infection.

## Materials and Methods

### Virus, insect vector, and plant samples

The sources of RDV-O and RDV-S were described previously [17]. Both strains were propagated and have been maintained in rice plants (*Oryza sativa* L. cv. Nipponbare) since 1984. For maintenance of RDV, rice plants were inoculated at the three- to four-leaf stage with a viruliferous green leafhopper (GLH: *Nephotettix cincticeps*) at least once a year. All rice plants were grown in the greenhouse, where temperatures fluctuated between 25 and 30°C in the spring to autumn.

In 1984, rice plants were inoculated with RDV-O. One to two months after inoculation, the virus, designated as D84, was purified according to the method described previously [65], and stored at −70°C. In 2006, the purified RDV-D84 was injected into instars of GLH and the insects were kept in a group for 10 to 14 days on healthy rice plants in a 28°C growth chamber. The insects were transferred to rice seedlings grown to the two- to three-leaf stage for inoculation of RDV-D84. The inoculated plants were placed in the greenhouse.

GLH were maintained in cages that contained rice seedlings in an insect-rearing room at 25–27°C. To obtain viruliferous GLH, nymphs were reared on virus-infected rice plants for 2 days, and insects were maintained up to the adult stage with occasional replacement of seedlings by healthy rice seedlings. Virus-free GLH were reared on healthy seedlings.

Fourteen seeds of *Oryza sativa* cv. Nipponbare, which is susceptible to RDV, were sown in a pot (85 mm in diameter and 75 mm in height) filled with about 250 ml of a commercial soil mixture (Bonsol, Sumitomo Chemical, Tokyo, Japan). The plants were grown under well-watered conditions in an air-conditioned greenhouse (25±3°C, natural sunlight). Fourteen seedlings at the two-leaf stage in a single pot were exposed to 70 viruliferous or virus-free (for mock inoculation) GLH in an inoculation chamber (34 cm wide by 26 cm deep by 34 cm high) for 24 h (25±3°C, continuous light conditions). After the insects were removed from the plants, the seedlings were placed in an air-conditioned greenhouse (25±3°C, natural sunlight). At 21 dpi, the shoots of the inoculated plants (except the meristem) were cut at 3 cm above the soil surface. After weighing of the samples, they were frozen in liquid nitrogen and stored at −80°C. After harvest, rice seedlings were grown continuously in the same greenhouse to evaluate virus infection. The experiment was repeated three times (three biological replicates). The heights of 20 rice plants infected with each RDV strain and 20 mock-inoculated plants were measured at 40 dpi. The significance of difference in plant heights was examined by ANOVA (P-value<0.01) and Fisher's least significant difference (LSD) test (LSD at 1% level).

### Detection and quantification of RDV

RDV infection and concentration were evaluated by the double antibody-sandwich enzyme-linked immunosorbent assay (DAS-ELISA) using an antiserum against RDV described previously [66]. To evaluate RDV infection, pieces (about 1 cm) of leaf sheath/stem tissue were harvested from each rice seedling and subjected to DAS-ELISA. To quantify the concentration of RDV in the rice plants, leaf samples were harvested from RDV-infected- and mock-inoculated plants at 30 dpi. After the leaf weight was measured, the samples were frozen in liquid nitrogen and stored at −80°C. The frozen samples were ground by a multibead shocker (MB501(S), YASUI KIKAI, Osaka, Japan) and were suspended with 10-fold weight of phosphate buffered saline (PBS) (10× extracts). The 10× extracts were further serially diluted between 2- and 2^8^-fold with PBS and subjected to DAS-ELISA. The concentration of the coat protein was estimated by comparing absorbance values of RDV-infected rice leaf saps with those of purified RDV of known concentrations at 410 nm. The significance of difference in virus concentrations among plants infected with the RDV strains was examined by ANOVA (P-value<0.05) and LSD test (LSD at 5% level).

### Sequencing of the RDV genome

Total RNA was extracted from RDV-infected rice plants using the RNeasy plant mini kit (Qiagen, Valencia, CA, USA) according to the manufacturer's instructions, and then reverse transcribed using SuperScript III (Invitrogen, Carlsbad, CA, USA) with random primers. The cDNA of the RDV genome was amplified by PCR using KOD DNA polymerase (TOYOBO, Osaka, Japan). The PCR protocol consisted of 1 min at 94°C, followed by 30 cycles of 15 s at 94°C, 15 s at 55°C, and 1 min at 68°C, and final extension time of 5 min at 68°C. PCR products of the expected size were purified by a PCR purification kit (Qiagen, Valencia, CA, USA) and directly sequenced in both directions using an ABI 3130 genetic analyzer with an ABI BigDye terminator v1.1 cycle sequencing kit (Applied Biosystems, Foster City, CA, USA). The nucleotide sequence data were compiled and analyzed with Genetyx-Win version 6 (Software Development, Tokyo, Japan).

### RNA extraction

Prior to RNA extraction, RDV infection in plants to be used for RNA extraction was examined by DAS-ELISA. For extraction of RNA from RDV-inoculated plants, we used only those confirmed to be infected with RDV. RNA samples were extracted from five independent plants in the same replicates by the RNeasy Maxi kit (Qiagen, Valencia, CA, USA). For this microarray experiment, we prepared 12 RNA samples (three RDV strains and one mock×three biological replicates). The concentration and quality of total RNA were examined by Nanodrop (Nanodrop ND-1000, Nanodrop Technologies, Wilmington, DE, USA) and BioAnalyzer (G2938A, Agilent Technologies, Santa Clara, CA, USA), respectively.

### Microarray experiment and data analysis

To analyze gene responses to RDV infection, we used a two-dye method, which directly compared expression profiles between two samples on the same microarray. The details of the microarray experiment and data analysis were described previously [8]. In brief, cyanine 3(Cy3)- or cyanine 5 (Cy5)-labeled complementary RNA (cRNA) samples were synthesized from 850 ng of the total RNA using the Low-input RNA labeling kit (Agilent Technologies, Santa Clara, CA, USA). In this study, RDV-infected and mock samples were labeled by Cy3 and Cy5, respectively. Hybridization solution was prepared with 825 ng each of Cy3- and Cy5-labeled cRNA preparations using the In situ hybridization kit plus (Agilent Technologies, Santa Clara, CA, USA). Hybridization and washing of microarray slides were performed following the manufacturer's protocols. After being washed, the slide image files were produced by the DNA microarray scanner (G2505B; Agilent Technologies, Santa Clara, CA, USA).

Signal intensities of Cy3 and Cy5 were extracted from the image files and normalized in each array by Feature Extraction version 9.5 (Agilent Technologies, Santa Clara, CA, USA). Signal intensities among all microarray data were normalized according to the quantile method (Global normalization) by EXPANDER ver. 4.1 [67]. A gene was declared “expressed” if the average signal intensity of the gene was higher than 6 in at least one condition; otherwise, the gene was considered not expressed. A DEG was defined as an expressed gene with 1) a log_2_-based ratio (RDV-inoculated sample/mock-inoculated sample) higher than 0.585 or lower than −0.585 and 2) significant changes in gene expression of P≤0.05 by a paired t-test (permutation: all, FDR collection: adjusted Bonferroni method). Data processing was performed using Mev version 4.4 [68]. The outputs of microarray analysis used in this study (series number GSE24937) are available at NCBI-GEO [69].

### RT-PCR

Complementary DNA (cDNA) fragments for transcripts of selected rice genes or the RDV genome were synthesized using 1,000 ng of the corresponding RNA with 50 ng/µl of random hexamer by SuperScript III reverse transcriptase (Invitrogen, USA). The resultant reaction mixtures containing cDNA were diluted four times. Some 4 µl of diluted mixture was used for PCR. Primers for rice genes were designed by Primer 3 [70]. Designed primers are shown in Supplementary Figure S1. The cycling program was initial denaturation for 2 min at 95°C, followed by 30 to 40 cycles of 15 s at 95°C, 15 s at variable annealing temperatures, and 45 s at 68°C, with a final extension of 1 min at 68°C (GeneAmp PCR System 9700; Applied Biosystems, USA). Annealing temperature was dependent on the Tm of the designed primers, and was between 50 and 60°C.

## Supporting Information

Figure S1 **Disease symptoms in plants infected with three RDV strains.** A): Rice plants stunted by infection with RDV strains at 30 dpi. Bar: 10 cm. B) Chlorotic stripes on leaf of an RDV-S-infected plant. Bar: 1 cm. C)
(TIF)

Figure S2 **DEGs evaluated by RT-PCR.** The numbers are the normalized signal intensity and log_2_-based differential expression ratios by microarray analysis. ns: log_2_-based differential expression ratio of the gene not significantly differentially expressed.
(TIF)

Figure S3 **Response in abiotic stress responsive gene families to RDV infection.** See Figure 4 for details.
(TIF)

Figure S4 **Response of genes for transcription factors involved in development and morphogenesis processes to RDV infection.** See Figure 4 for details.
(TIF)

Figure S5 **Response of genes whose products localized in cell wall to RDV infection.** See Figure 4 for details.
(TIF)

Figure S6 **Response of genes associated with photosynthesis-, and carbon fixation-related processes to RDV infection.** See Figure 4 for details.
(TIF)

Table S1 **Characterization of three RDV strains.** A: raw data of plant height, B: nucleotide sequences of 12 segments, C: amino acid sequences of 12 proteins
(XLS)

Table S2 **The list of DEGs.**
(XLS)

## References

[1] Dardick C (2007). Comparative expression profiling of *Nicotiana benthamiana* leaves systemically infected with three fruit tree viruses.. Mol Plant-Microbe Interact.

[2] Senthil G, Liu H, Puram VG, Clark A, Stromberg A (2005). Specific and common changes in *Nicotiana benthamiana* gene expression in response to infection by enveloped viruses.. J Gen Virol.

[3] Havelda Z, Várallyay E, Válóczi A, Burgyán J (2008). Plant virus infection-induced persistent host gene downregulation in systemically infected leaves.. Plant J.

[4] Babu M, Griffiths JS, Huang TS, Wang A (2008). Altered gene expression changes in *Arabidopsis* leaf tissues and protoplasts in response to *Plum pox virus* infection.. BMC Genomics.

[5] Agudelo-Romero P, Carbonell P, Perez-Amador MA, Elena SF (2008). Virus adaptation by manipulation of host's gene expression.. PLoS One.

[6] Catoni M, Miozzi L, Fiorilli V, Lanfranco L, Accotto GP (2009). Comparative analysis of expression profiles in shoots and roots of tomato systemically infected by *Tomato spotted wilt virus* reveals organ-specific transcriptional responses.. Mol Plant-Microbe Interact.

[7] Shimizu T, Satoh K, Kikuchi S, Omura T (2007). The repression of cell wall- and plastid-related genes and the induction of defense-related genes in rice plants infected with *Rice dwarf virus*.. Mol Plant-Microbe Interact.

[8] Satoh K, Kondoh H, Sasaya T, Shimizu T, Choi IR (2010). Selective modification of rice (*Oryza sativa*) gene expression by *Rice stripe virus* infection.. J Gen Virol.

[9] Díaz-Pendón JA, Ding SW (2008). Direct and indirect roles of viral suppressors of RNA silencing in pathogenesis.. Annu Rev Phytopathol.

[10] Nagasaki H, Itoh J, Hayashi K, Hibara K, Satoh-Nagasawa N (2007). The small interfering RNA production pathway is required for shoot meristem initiation in rice.. Proc Natl Acad Sci USA.

[11] Yu D, Fan B, MacFarlane SA, Chen Z (2003). Analysis of the involvement of an inducible *Arabidopsis* RNA-dependent RNA polymerase in antiviral defense.. Mol Plant-Microbe Interact.

[12] Vaucheret H (2008). Plant ARGONAUTES.. Trends Plant Sci.

[13] Liu D, Raghothama KG, Hasegawa PM, Bressan RA (1994). Osmotin overexpression in potato delays development of disease symptoms.. Proc Natl Acad Sci U S A.

[14] Banerjee J, Maiti MK (2010). Functional role of rice germin-like protein1 in regulation of plant height and disease resistance.. Biochem Biophys Res Commun.

[15] Stotz HU, Spence B, Wang Y (2009). A defensin from tomato with dual function in defense and development.. Plant Mol Biol.

[16] Hibino H (1996). Biology and epidemiology of rice viruses.. Annu Rev Phytopathol.

[17] Kimura I, Minobe Y, Omura T (1987). Changes in a nucleic acid and a protein component of *Rice dwarf virus* particles associated with an increase in symptom severity.. J Gen Virol.

[18] Xie Z, Johansen LK, Gustafson AM, Kasschau KD, Lellis AD (2004). Genetic and functional diversification of small RNA pathways in plants.. PLoS Biol.

[19] Sánchez G, Gerhardt N, Siciliano F, Vojnov A, Malcuit I (2010). Salicylic acid is involved in the Nb-mediated defense responses to *Potato virus X* in *Solanum tuberosum*.. Mol Plant-Microbe Interact.

[20] Takahashi H, Kanayama Y, Zheng MS, Kusano T, Hase S (2004). Antagonistic interactions between the SA and JA signaling pathways in *Arabidopsis* modulate expression of defense genes and gene-for-gene resistance to *Cucumber mosaic virus*.. Plant Cell Physiol.

[21] Lee MW, Qi M, Yang Y (2001). A novel jasmonic acid-inducible rice *myb* gene associates with fungal infection and host cell death.. Mol Plant-Microbe Interact.

[22] Ye H, Du H, Tang N, Li X, Xiong L (2009). Identification and expression profiling analysis of TIFY family genes involved in stress and phytohormone responses in rice.. Plant Mol Biol.

[23] Love AJ, Laval V, Geri C, Laird J, Tomos AD (2007). Components of *Arabidopsis* defense- and ethylene-signaling pathways regulate susceptibility to *Cauliflower mosaic virus* by restricting long-distance movement.. Mol Plant-Microbe Interact.

[24] Fischer U, Dröge-Laser W (2004). Overexpression of NtERF5, a new member of the tobacco ethylene response transcription factor family, enhances resistance to *Tobacco mosaic virus*.. Mol Plant-Microbe Interact.

[25] Qiu D, Xiao J, Ding X, Xiong M, Cai M (2007). OsWRKY13 mediates rice disease resistance by regulating defense-related genes in salicylate- and jasmonate-dependent signaling.. Mol Plant-Microbe Interact.

[26] Shimono M, Sugano S, Nakayama A, Jiang CJ, Ono K (2007). Rice WRKY45 plays a crucial role in benzothiadiazole-inducible blast resistance.. Plant Cell.

[27] van Loon LC, Rep M, Pieterse CM (2006). Significance of inducible defense-related proteins in infected plants.. Annu Rev Phytopathol.

[28] Sakamoto T, Miura K, Itoh H, Tatsumi T, Ueguchi-Tanaka M (2004). An overview of gibberellin metabolism enzyme genes and their related mutants in rice.. Plant Physiol.

[29] Hirsch S, Oldroyd GE (2009). GRAS-domain transcription factors that regulate plant development.. Plant Signal Behav.

[30] Zhao Y (2010). Auxin biosynthesis and its role in plant development.. Annu Rev Plant Biol.

[31] Guilfoyle TJ, Hagen G (2007). Auxin response factors.. Curr Opin Plant Biol.

[32] Jain M, Tyagi AK, Khurana JP (2006). Genome-wide analysis, evolutionary expansion, and expression of early auxin-responsive SAUR gene family in rice (*Oryza sativa*).. Genomics.

[33] van der Graaff E, Laux T, Rensing SA (2009). The WUS homeobox-containing (WOX) protein family.. Genome Biol.

[34] Park SH, Kim CM, Je BI, Park SH, Park SJ (2007). A *Ds*-insertion mutant of *OSH6* (*Oryza sativa* Homeobox 6) exhibits outgrowth of vestigial leaf-like structures, bracts, in rice.. Planta.

[35] Abiko M, Ohmori Y, Hirano HY (2008). Genome-wide expression profiling and identification of genes under the control of the *DROOPING LEAF* gene during midrib development in rice.. Genes Genet Syst.

[36] Wang S, Chang Y, Guo J, Chen JG (2007). *Arabidopsis* Ovate Family Protein 1 is a transcriptional repressor that suppresses cell elongation.. Plant J.

[37] Martín-Trillo M, Cubas P (2010). TCP genes: a family snapshot ten years later.. Trends Plant Sci.

[38] Shigyo M, Tabei N, Yoneyama T, Yanagisawa S (2006). Evolutionary processes during the formation of the plant-specific Dof transcription factor family.. Plant Cell Physiol.

[39] Shikata M, Koyama T, Mitsuda N, Ohme-Takagi M (2009). *Arabidopsis* SBP-box genes *SPL10*, *SPL11* and *SPL2* control morphological change in association with shoot maturation in the reproductive phase.. Plant Cell Physiol.

[40] Wang L, Yin H, Qian Q, Yang J, Huang C (2009). NECK LEAF 1, a GATA type transcription factor, modulates organogenesis by regulating the expression of multiple regulatory genes during reproductive development in rice.. Cell Res.

[41] Mao C, Ding W, Wu Y, Yu J, He X (2007). Overexpression of a NAC-domain protein promotes shoot branching in rice.. New Phytol.

[42] Zhang S, Chen C, Li L, Meng L, Singh J (2005). Evolutionary expansion, gene structure, and expression of the rice wall-associated kinase gene family.. Plant Physiol.

[43] Wang XB, Wu Q, Ito T, Cillo F, Li WX (2009). RNAi-mediated viral immunity requires amplification of virus-derived siRNAs in *Arabidopsis thaliana*.. Proc Natl Acad Sci USA.

[44] Kapoor M, Arora R, Lama T, Nijhawan A, Khurana JP (2008). Genome-wide identification, organization and phylogenetic analysis of Dicer-like, Argonaute and RNA-dependent RNA Polymerase gene families and their expression analysis during reproductive development and stress in rice.. BMC Genomics.

[45] Yoshii M, Yamazaki M, Rakwal R, Kishi-Kaboshi M, Miyao A (2010). The NAC transcription factor RIM1 of rice is a new regulator of jasmonate signaling.. Plant J.

[46] Yoshii M, Shimizu T, Yamazaki M, Higashi T, Miyao A (2009). Disruption of a novel gene for a NAC-domain protein in rice confers resistance to *Rice dwarf virus*.. Plant J.

[47] Babu M, Gagarinova AG, Brandle JE, Wang A (2008). Association of the transcriptional response of soybean plants with soybean mosaic virus systemic infection.. J Gen Virol.

[48] Margis-Pinheiro M, Zhou XR, Zhu QH, Dennis ES, Upadhyaya NM (2005). Isolation and characterization of a *Ds*-tagged rice (*Oryza sativa* L.) GA-responsive dwarf mutant defective in an early step of the gibberellin biosynthesis pathway.. Plant Cell Rep.

[49] Rieu I, Ruiz-Rivero O, Fernandez-Garcia N, Griffiths J, Powers SJ (2008). The gibberellin biosynthetic genes AtGA20ox1 and AtGA20ox2 act, partially redundantly, to promote growth and development throughout the *Arabidopsis* life cycle.. Plant J.

[50] Ikeda A, Ueguchi-Tanaka M, Sonoda Y, Kitano H, Koshioka M (2001). *slender* rice, a constitutive gibberellin response mutant, is caused by a null mutation of the SLR1 gene, an ortholog of the height-regulating gene GAI/RGA/RHT/D8.. Plant Cell.

[51] Dill A, Sun T (2001). Synergistic derepression of gibberellin signaling by removing RGA and GAI function in *Arabidopsis thaliana*.. Genetics.

[52] Lo SF, Yang SY, Chen KT, Hsing YI, Zeevaart JA (2008). A novel class of gibberellin 2-oxidases control semidwarfism, tillering, and root development in rice.. Plant Cell.

[53] Attia KA, Abdelkhalik AF, Ammar MH, Wei C, Yang J (2009). Antisense phenotypes reveal a functional expression of OsARF1, an auxin response factor, in transgenic rice.. Curr Issues Mol Biol.

[54] Lim PO, Lee IC, Kim J, Kim HJ, Ryu JS (2010). Auxin response factor 2 (ARF2) plays a major role in regulating auxin-mediated leaf longevity.. J Exp Bot.

[55] Elhiti M, Stasolla C (2009). Structure and function of homodomain-leucine zipper (HD-Zip) proteins.. Plant Signal Behav.

[56] Takasaki H, Maruyama K, Kidokoro S, Ito Y, Fujita Y (2010). The abiotic stress-responsive NAC-type transcription factor OsNAC5 regulates stress-inducible genes and stress tolerance in rice.. Mol Genet Genomics.

[57] Wasternack C (2007). Jasmonates: an update on biosynthesis, signal transduction and action in plant stress response, growth and development.. Ann Bot.

[58] Anand A, Schmelz EA, Muthukrishnan S (2003). Development of a lesion-mimic phenotype in a transgenic wheat line overexpressing genes for pathogenesis-related (PR) proteins is dependent on salicylic acid concentration.. Mol Plant-Microbe Interact.

[59] Cao X, Zhou P, Zhang X, Zhu S, Zhong X (2005). Identification of an RNA silencing suppressor from a plant double-stranded RNA virus.. J Virol.

[60] Selth LA, Randles JW, Rezaian MA (2004). Host responses to transient expression of individual genes encoded by *Tomato leaf curl virus*.. Mol Plant-Microbe Interact.

[61] Szilassy D, Salánki K, Balázs E (1999). Stunting induced by cucumber mosaic cucumovirus-infected *Nicotiana glutinosa* is determined by a single amino acid residue in the coat protein.. Mol Plant-Microbe Interact.

[62] Omura T, Mertens PPPC, Fauquet CM, Mayo MA, Maniloff J, Desselberger U, Ball AL (2005). Phytoreovirus.. Virus Taxonomy: eighth report of the International Committee on Taxonomy of Viruses.

[63] Li Y, Bao YM, Wei CH, Kang ZS, Zhong YW (2004). *Rice dwarf phytoreovirus* segment S6-encoded nonstructural protein has a cell-to-cell movement function.. J Virol.

[64] Zhu S, Gao F, Cao X, Chen M, Ye G (2005). The rice dwarf virus P2 protein interacts with ent-kaurene oxidases in vivo, leading to reduced biosynthesis of gibberellins and rice dwarf symptoms.. Plant Physiol.

[65] Kimura I, Kodama T, Suzuki N (1968). Customization of a method for *Rice dwarf virus* purification and activity of purified virus.. Ann Phytopathol Soc Jpn.

[66] Takahashi Y, Omura T, Shohara K, Tsuchizaki T (1991). Comparison of four serological methods for practical detection of ten viruses of rice in plants and insects.. Plant Dis.

[67] Ulitsky I, Maron-Katz A, Shavit S, Sagir D, Linhart C (2010). Expander: from expression microarrays to networks and functions.. Nat Protoc.

[68] Saeed AI, Bhagabati NK, Braisted JC, Liang W, Sharov V (2006). TM4 microarray software suite.. Methods Enzymol.

[69] Edgar R, Domrachev M, Lash AE (2002). Gene Expression Omnibus: NCBI gene expression and hybridization array data repository.. Nucleic Acids Res.

[70] Rozen S, Skaletsky HJ, Krawetz S, Misener S (2000). Primer3 on the WWW for general users and for biologist programmers.. Bioinformatics Methods and Protocols: Methods in Molecular Biology.

