# Supplementary figures and images for: Relationship between Symptoms and Gene Expression Induced by the Infection of Three Strains of Rice dwarf virus
Source: PLoS One. 2011 Mar 22;6(3):e18094. doi: 10.1371/journal.pone.0018094 (PMC3062569; doi:10.1371/journal.pone.0018094)

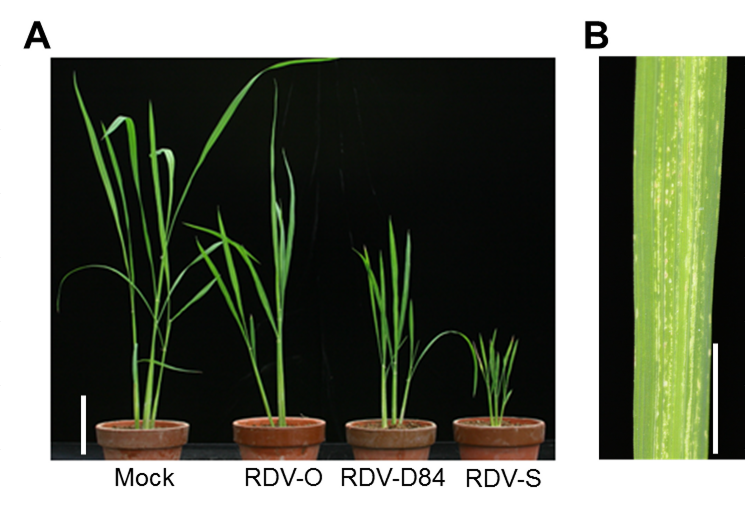

Supplement: Figure S1 — Disease symptoms in plants infected with three RDV strains. A): Rice plants stunted by infection with RDV strains at 30 dpi. Bar: 10 cm. B) Chlorotic stripes on leaf of an RDV-S-infected plant. Bar: 1 cm. C) (TIF) [file pone.0018094.s001.tif]

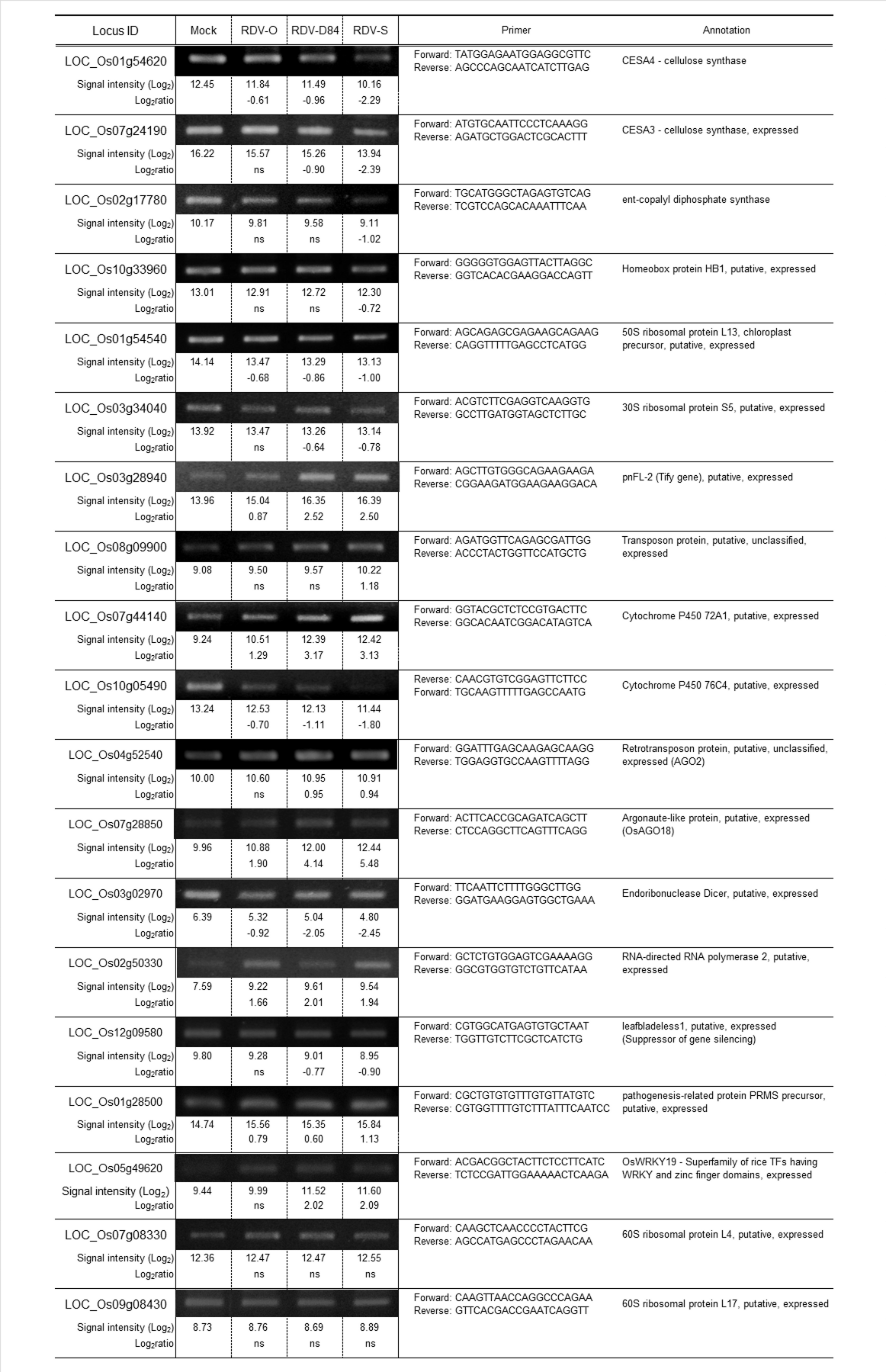

Supplement: Figure S2 — DEGs evaluated by RT-PCR. The numbers are the normalized signal intensity and log2-based differential expression ratios by microarray analysis. ns: log2-based differential expression ratio of the gene not significantly differentially expressed. (TIF) [file pone.0018094.s002.tif]

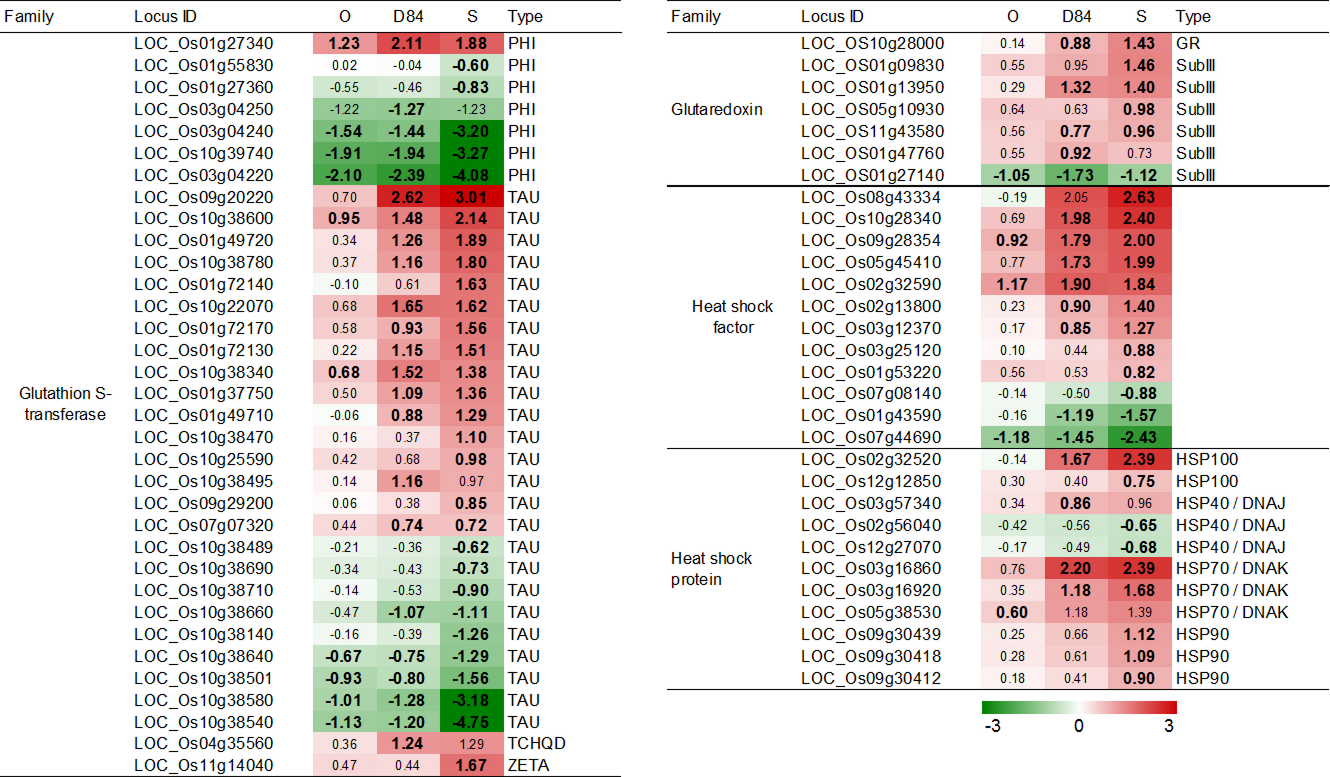

Supplement: Figure S3 — Response in abiotic stress responsive gene families to RDV infection. See Figure 4 for details. (TIF) [file pone.0018094.s003.tif]

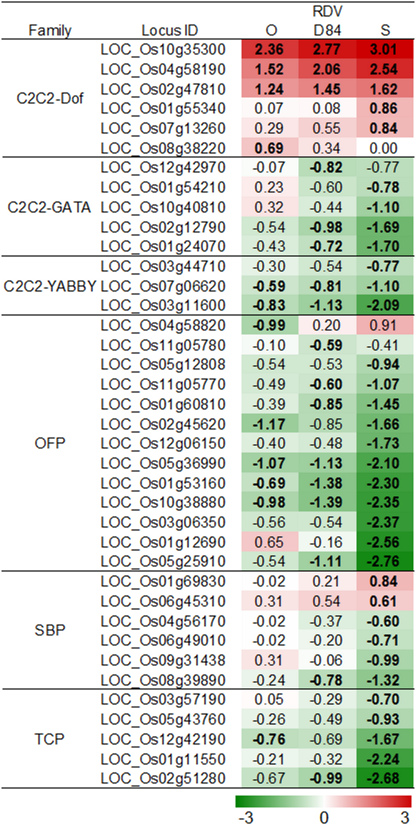

Supplement: Figure S4 — Response of genes for transcription factors involved in development and morphogenesis processes to RDV infection. See Figure 4 for details. (TIF) [file pone.0018094.s004.tif]

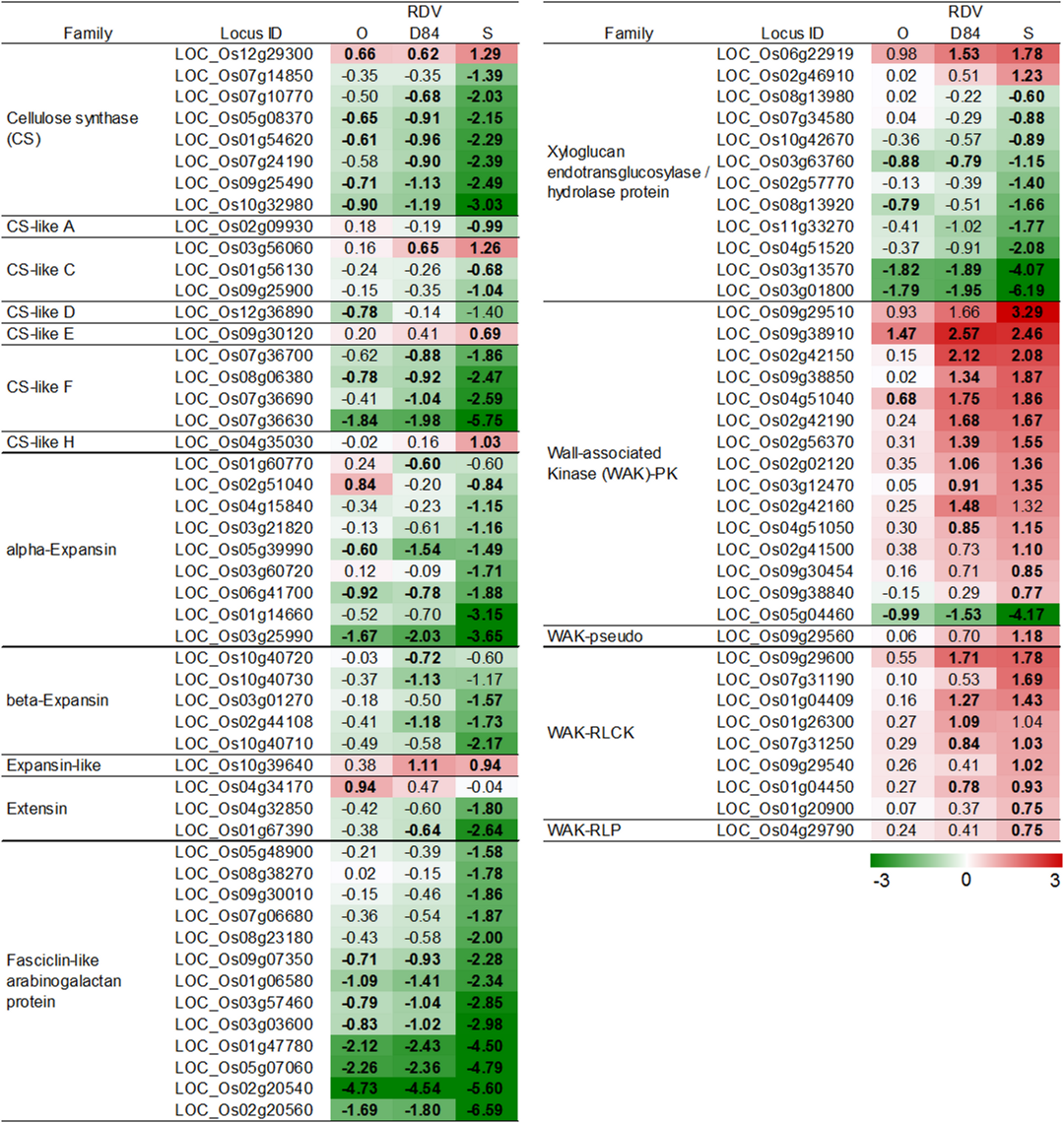

Supplement: Figure S5 — Response of genes whose products localized in cell wall to RDV infection. See Figure 4 for details. (TIF) [file pone.0018094.s005.tif]

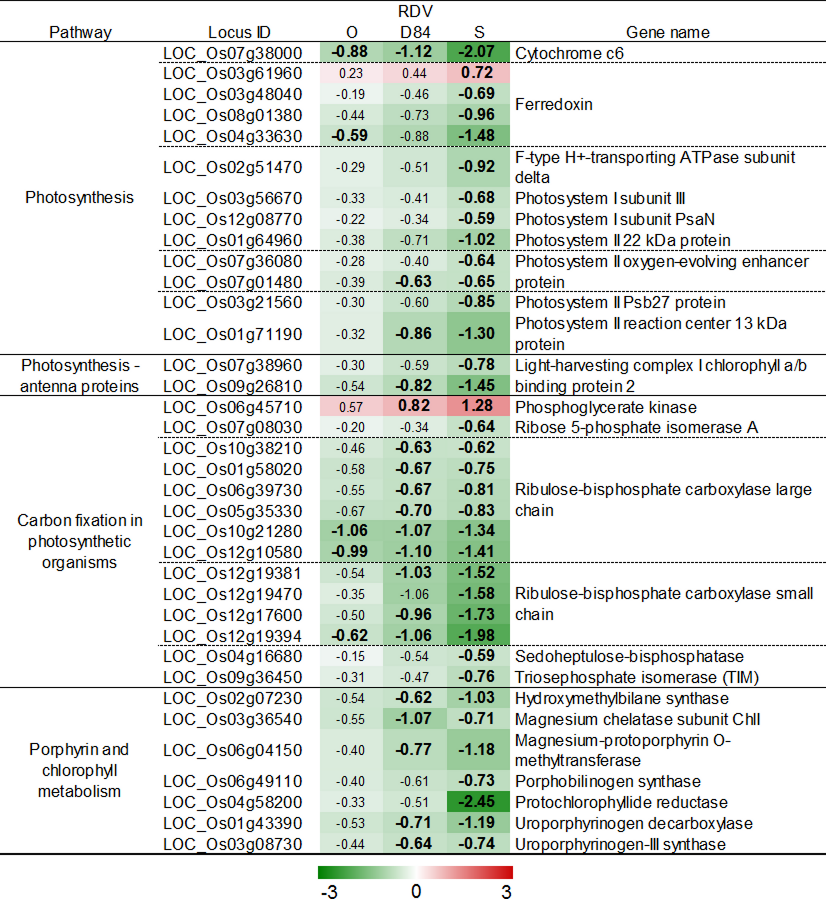

Supplement: Figure S6 — Response of genes associated with photosynthesis-, and carbon fixation-related processes to RDV infection. See Figure 4 for details. (TIF) [file pone.0018094.s006.tif]
